# Estimating Disease Heritability from Electronic Healthcare Records: A Proof-of-Concept Study.

**DOI:** 10.23889/ijpds.v7i3.1951

**Published:** 2022-08-25

**Authors:** Lisa Lix, Amani Hamad, Lin Yan, Joseph A. Delaney, Elizabeth Wall-Wieler, Mohammad Jafari Jozani, Shantanu Banerji, Olawale Ayilara, Pingzhao Hu

**Affiliations:** 1 University of Manitoba; 2 CancerCare Manitoba

## Objectives

A family history of a chronic disease often predicts disease risk, with predictive value determined by heritability, the proportion of variation in risk explained by inherited genetic factors. Our objective was to assess the validity of disease heritability estimates from electronic healthcare records (EHRs) that capture family relationships and disease diagnoses.

## Approach

A population-based investigation was conducted using healthcare records from Manitoba, Canada for 1970 to 2021. We constructed family relationships for up to four generations using health insurance registration information containing unique family and individual identifiers. Health histories for family members were created using diagnosis codes in hospital and physician visit records. Linear mixed-effects models were used to estimate heritability (h) for 130 chronic health conditions using open-source Clinical Classifications Software that defines clinically-meaningful disease categories. Comparisons between EHR-derived estimates and genetically-derived estimates from published studies were used to assess validity of the methodology.

## Results

Health insurance registration data were used to construct 10,000 families that included 116,879 individuals. Median family size was 9 (interquartile range: 8). Median observation time was 39.6 years (interquartile range: 25.7). Males comprised half (51.0%) of family members. A total of 272,114 familial relationships were identified; slightly more than half (53%) were first degree (i.e., child and parent) relationships. One-third (33.2%) of families were comprised of four generations; only 15.3% were comprised of two generations. Heritability estimates were consistent with published genetically-derived estimates for several conditions, including diabetes (EHR h = 0.29 vs. 0.22), anemia (EHR h = 0.21 vs. 0.20), and asthma (EHR h = 0.34 vs. 0.33). However, inconsistencies were identified for pancreatic disorders, gastrointestinal conditions, some mental health conditions, and heart disease.

## Conclusion

EHRs provide a promising approach to explore heritability of selected health conditions in large, diverse populations. Inconsistencies between EHR-derived and genetically-derived estimates are indicative of the limitations of diagnoses recorded for administrative purposes. Future research will explore sex-specific heritability estimates and effects of change in disease diagnosis coding over time.

